# Imagination and the creative process: a systematic review

**DOI:** 10.3389/fpsyg.2026.1686856

**Published:** 2026-07-03

**Authors:** Kristen N. Lamb, Jeb S. Puryear

**Affiliations:** 1 University of Alabama, Tuscaloosa, United States; 2 University of Montana, Missoula, United States

**Keywords:** creative process, creativity, imagination, mixed method, systematic review

## Abstract

Imagination is an essential component of the creativity process. Despite its important role in the creative process, imagination has received comparatively little attention in creativity research. The purpose of this systematic review was to examine how imagination is defined in empirical literature, the role it plays in creativity, and how these findings can inform an evidence-based definition. Using an integrated mixed-methods research synthesis, 42 peer-reviewed studies published between 2012 and 2024 were analyzed across disciplines, populations, and methodological approaches. Clear thematic patterns revealed how imagination is conceptualized and supported imagination and creativity as related but distinct constructs. Based on the findings of this review, researchers propose a working definition of imagination, discuss implications, and highlight areas where additional research is needed.

## 1 Introduction

Imagination has been referred to as the “magic and mystery” of the creative process and product (Khatena, 1975, p. 149), and others have similarly characterized imagination as a “gift to humans” (Liang et al., 2012, p. 1037) or a gift from the Muses (Piirto, 2000). Vygotsky (2004) described imagination as the foundation for all creative processes, asserting its importance in “all aspects of cultural life, enabling artistic, scientific, and technical creation alike” (p. 9). Liang and Chia (2014) described imagination as an advanced cognitive ability and a promoter of creativity.

Although descriptions of imagination vary, one consistent idea throughout the literature is the connection between imagination and creativity (Khatena, 1975; Liang and Chia, 2014; Liang and Lin, 2015; Lin and Tsau, 2013). These two constructs work in concert with one another (Pelaprat and Cole, 2011; Vygotsky, 2004), and although related, imagination and creativity are often viewed as sequential, yet dependent processes. In other words, imagination is the fuel and creativity—the wheels, both necessary in the generating of novel ideas and setting those ideas into motion. Discussions of the connection between imagination and the creative process are ubiquitous; however, this connection is often illustrated through varied conceptual and philosophical works as well as in everyday discourse. In this study, we aim to synthesize and distill those illustrations to deliver a more consensus-based view of imagination's role in the creative process.

### 1.1 Imagination: instrumental, yet often forgotten, component of creativity

Saturated by information and technology, today's problems can only be solved with creative thinking (Hennessey and Amabile, 2010; Olszewski-Kubilius et al., 2016). Additionally, creativity is a skill necessary in developing the ability to break away from one-track mindsets (Dai and Cheng, 2017) and dogmatic thinking (Olszewski-Kubilius et al., 2016). Further, creativity is associated with lifelong success (Hong and Ditzler, 2013; Plucker et al., 2004) connected to individuals' social and emotional wellness as well as academic (Plucker et al., 2004) and entrepreneurial/organizational success (Amabile, 1988; Hennessey and Amabile, 2010). Most of these studies focus on the role of creativity, yet rarely do these studies address the role imagination plays in the creative process (Forgeard and Kaufman, 2016; Liang et al., 2012). Neglecting the examination of imagination in empirical research and applied settings leaves an incomplete understanding of the role of creativity in the advancement of society.

Imagination is an important part of creative thinking and involves two main processes: (a) generative, or processes that rely on imagination to generate ideas; and (b) exploratory, or processes in which multiple interpretations of generated ideas are examined or explored (Finke, 1996; Gotlieb et al., 2019). Imagination includes multiple cognitive processes such as reflection, pretend play, perspective-taking, identity formation, and mind wandering (Abraham, 2016; Gotlieb et al., 2019; Runco and Pina, 2013). Despite some apparent overlap between how imagination and creativity (e.g., generating original ideas) are conceptualized and discussed in the literature, creativity differs in that it should also be useful and intentional (Gotlieb et al., 2019; Runco and Pina, 2013).

Although creativity research is robust, dating beyond Guilford's 1950 APA presidential address, research focused on imagination and the role it plays in the creative process remains hazy (Liang et al., 2013). Further, empirical studies that examine and measure the construct of imagination are lacking (Liang et al., 2012). To address this issue, Liang and colleagues conducted a research synthesis in which they evaluated research from 1900 to 2012 with two aims: (a) to clarify the term imagination, and (b) to identify associated markers of imagination. From their work, they identified two dimensions and ten markers of imagination. The two dimensions included creative imagination (indicated by novelty, productivity, exploration, focus, intuition, and sensibility) and reproductive imagination (indicated by dialectics, crystallization, transformation, and effectiveness). The two dimensions of imagination are distinct in that creative imagination is typically associated with originality and characterized by groundbreaking discoveries and revolutions, whereas reproductive imagination is typically associated with reproducing mental images and characterized by exploration, focus, intuition, novelty, sensibility, and productivity (Liang et al., 2012). Through confirmatory factor analysis, their results supported the two dimensions of imagination.

In 2016, Forgeard and Kaufman reviewed 200 randomly selected peer-reviewed articles to examine who studies imagination, creativity, and innovation, as well as whether researchers addressed the importance of the topic, and what those reasons were. Their results suggested that the construct of creativity was most represented in creativity, education, and psychology journals, and innovation was most represented in business and industrial/organization journals. Very few studies (4%) investigated the construct of imagination. Perhaps more startling, 71% of the reviewed articles provided minimal or no explanation addressing why individuals should care about imagination, creativity, and/or innovation (though they did account for potential limiting factors, such as space limitations and readership associated with certain journals). Forgeard and Kaufman concluded by placing an urgent call for research that uses more explicit language that moves readers beyond the assumption that creativity is beneficial and instead clearly states when, how, for whom, and why it is beneficial.

There is no question that imagination and creativity are linked, and the extensive scholarship in creativity has afforded a robust, empirically rooted understanding of creativity. Imagination, on the other hand, has not. With this review, we aimed to respond to Forgeard and Kaufman's (2016, p. 256) call to “do better” by developing explicit research questions to improve our understanding of imagination as a construct and its role in the creative process.

## 2 Review objectives and research questions

The systematic review of scholarly work in this area is essential in expanding our understanding of imagination as a construct and its role in the creative process. Our specific aim was to clarify how imagination is defined and described in the literature and examine its role within creativity, including common correlates. The following research questions guided this review:

1. How is imagination defined as a construct in empirical studies related to creativity?

(1a) Do the authors define imagination in the paper (explicit, implicit, not at all)?
(1b) What is the level of agreement or consistency of keywords used to define or describe imagination?

2. What role does imagination play in the creative process?

(2a) What variables are most commonly used to investigate this relationship?

3. Based on the emerging themes in authors' definitions of imagination (RQ1) and its role in the creative process (RQ2), what definition best synthesizes empirically grounded characteristics of imagination and captures its role in the creative process? (synthesizes RQ1 and RQ2).

## 3 Method

This review expands on prior work from other scholars' reviews on the topic of imagination (Forgeard and Kaufman, 2016; Liang et al., 2012). The design used for this systematic review was an integrated mixed method research synthesis. Both authors of this review are creativity researchers with experience conducting systematic reviews (e.g., Mullet et al., 2016; Puryear and Lamb, 2020). Search parameters and eligibility criteria were discussed prior to beginning the search and screening process.

### 3.1 Search strategy

After informally reviewing relevant literature, the list of search terms was created. We used free-text words and Boolean operators (Heyvaert et al., 2017). We also used truncation where possible to capture keyword variants. The term “imaginat^*^” was used as the primary search term, limited to the title or abstract, in order to identify articles in which imagination was a central focus of the journal article. We also used the key term “creativ^*^,” limited to the title or abstract, to narrow our search for studies with a focus on both creativity and imagination. For larger databases (e.g., Academic Search Premier), we added the term “method,” extended to the full text, to limit the pull of papers such as commentaries, interviews, etc. The electronic databases included in this search were Academic Search Premier, APA PsychINFO, and Google Scholar. We also used our key terms to electronically search titles and abstracts of the following journals based on relevancy to the topic: *Creativity Research Journal (CRJ)*; *Creativity-Theory-Research-Application (CTRA)*; *Imagination, Cognition, and Personality (ICAP); Journal of Creativity (JOC)*; *The Journal of Creative Behavior (JOCB)*; and *Thinking Skills and Creativity (TSC)*. “When and where possible,” for all databases and journals searched, “we used electronic information provided in the abstracts, instead of reading through all of the retrieved articles” (Heyvaert et al., 2013, p. 304).

**Figure 1:**
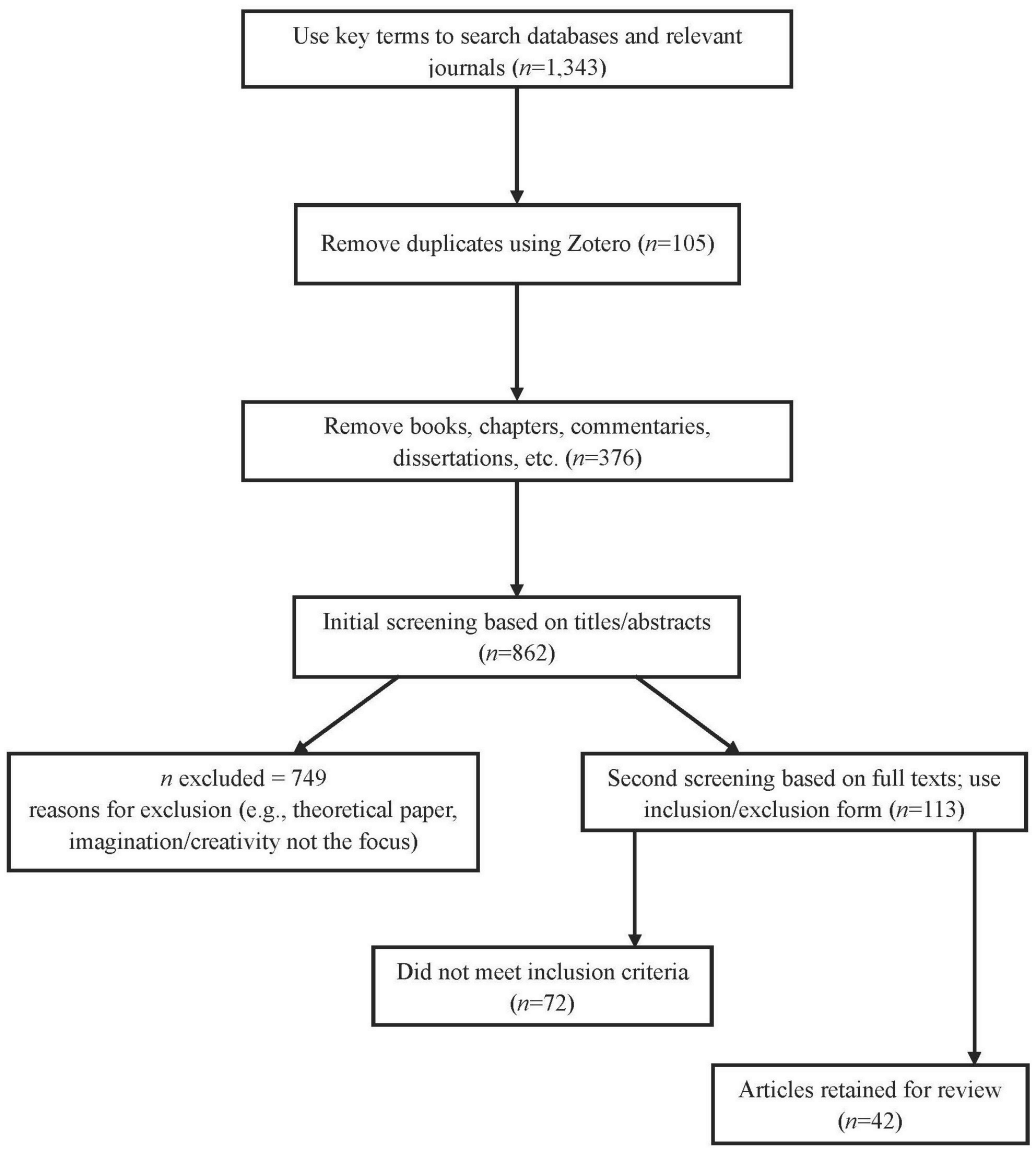
Selective sampling strategy. One article was identified as relevant to this review during the full-text screening phase.

The selective sampling strategy was used in this review as we wanted to “identify all relevant studies but only within specified limits” (Heyvaert et al., 2017, p. 74). For instance, Liang et al. (2012) summarized factors of imagination through a systematic review of the literature published between 1900 and 2012; for that reason, the dates in this study were limited to extend upon prior findings and capture the most recent decade (January 1, 2012 – December 31, 2024). Other limiters were applied to our initial search including peer-reviewed, empirical studies in scholarly journals that were published in the English language, and we used equivalent search strings and limitations for each database. See Figure 1 for an outline of our selective sampling strategy.

The first reviewer performed the search process by combining search terms with Boolean operators and adjusting the search string and limiters, as necessary, based on what the database or journal permitted. For instance, in the database Academic Search Premier, we used the limiters full text, peer-reviewed, publication date (2012–2024), document type article, English language, source type academic journal whereas when searching the database APA PsychInfo, we used peer-reviewed, exclude dissertations, academic journals, fully published, population group human, document type journal article. We made similar adjustments when searching each journal. After each search was conducted for each database or journal, the review author recorded the number of identified articles. The second reviewer followed the search parameters for every database and journal (*n* = 9) to ensure replicability. Of these, the second reviewer was able to replicate eight of the nine searches (89%). The exception was in the Academic Search Premier database. One reviewer had access to Academic Search Premier whereas the other reviewer had access to Academic Search Complete. Through the initial search, we identified 1,343 studies of which 105 were duplicates. After removing duplicates, we were left with 1,238 manuscripts. We then removed manuscripts that clearly did not meet our criteria, such as books, book chapters, and dissertations which left a total of 862 articles to move to the screening process. See Table 1 for a full list of search parameters and initial results for each database and journal.

**Table 1:** Search parameters and initial results.

Search terms	Database or journal	Search limiters	Hits
[title, abstract] imaginat^*^ AND [title, abstract] creativ^*^AND [all text] method	Academic Search Premier	Full Text	443
		Peer-Reviewed	
		Publication Type: Periodical	
		Document Type: Article	
		Source Type: Academic Journals	
		No expanders; find all search terms	
[AB] imaginat^*^ AND [AB] creativ^*^ AND [TX] method	APA PsychInfo	Peer-Reviewed	176
		Exclude Dissertations	
		Academic Journals	
		Fully Published	
		Population Group: Human	
		Document Type: Journal Article	
Imaginative imagination	*Creativity–Theory–Research–Application ^*a*^*		1
			0
[all in title] imaginative OR imagination AND creative OR creativity	Google Scholar	Exclude case law, citations, patents	599
imaginat^*^ [abstract] AND creativ^*^ [abstract]	Sage Journals: *Imagination, Cognition, and Personality*	Research Article	6
[title, abstract, keywords] imaginative	ScienceDirect: *JournalofCreativity*^b^		0
[title, abstract, keywords] imagination			2
[title, abstract, keywords] imaginative	ScienceDirect: *ThinkingSkillsandCreativity*^b^		21
[title, abstract, keywords] imagination			58
[Abstract: imaginat^*^] AND [Abstract: creativ^*^]	Taylor and Francis Online: *Creativity Research Journal*	Article Type: Article	18
		In Journal: Creativity Research Journal	
[abstract] imaginat^*^ AND [abstract] creativ^*^	WILEY Online Library: *TheJournalofCreativeBehavior*^b^		19
		Initial total	1,343
		Duplicates removed	105
		Final total	1,238

Unless otherwise specified, advanced search was used, and all searches were limited to publications in English between the dates January 1, 2012 and December 31, 2024.

^a^Journal does not permit any additional limiters beyond a single key term.

^b^Journal does not permit any additional limiters beyond key terms and publication dates.

### 3.2 Screening process and data extraction

During the initial screening, titles and abstracts for identified studies (*n* = 862) were reviewed by the first author to further exclude irrelevant studies. In many of these instances, the key terms were minimally mentioned in the abstract and clearly not related to the purpose of this review (i.e., imagination and creativity were not primary variables in the investigation). Excluded articles and the reason for exclusion were noted in an excel spreadsheet. To assess intercoder agreement for the initial screening phase, all titles and abstracts of identified studies from one database and one journal chosen at random were screened by the second reviewer with an initial agreement rate of 88%. Articles in question were discussed and from there reviewers reached consensus with a 99% agreement. From this screening, 113 articles were retained for a secondary screening (full-text review), and full-text copies of potentially relevant studies were retrieved and uploaded to Zotero as necessary (i.e., some full-text copies automatically exported during the initial search phase).

Next, the second screening phase was conducted, and additional inclusion criteria were applied to help narrow down the list of studies identified from the initial search and abstract/title screening to assess fitness of purpose (Heyvaert et al., 2017). First, the study had to be empirical with a clear methods section (quantitative, qualitative, mixed method) and include a description of the sample used in the study. Second, the relationship between imagination and creativity had to be addressed in the investigation. For example, during the screening process, one article was excluded as its central focus was on imagination and maladaptive personality traits rather than creativity (e.g., Castillo and Condon, 2024). Any disagreements were discussed until consensus was achieved. After full-text screening, 42 articles were identified as relevant and retained for this review (see Figure 1). Eligibility criteria were specified *a priori*. The screening form can be found in Appendix A.

Data was extracted according to the phenomenon of interest and the research questions guiding this review. For each article in the final sample, descriptive data was extracted (e.g., study design, participants, geographical location). To address the first research question, the following questions were used to guide coding: (a) Do the authors define imagination in the paper (explicit, implicit, not at all)? and (b) If explicitly or implicitly defined, how is imagination defined and what are the keywords used? To address the second research question, the following questions guided our coding: (a) What role does imagination play in the creative process? and (b) What are the variables used to investigate this relationship? A detailed version of our data extraction form is provided in Appendix B. We then examined prevalent thematic patterns, including frequencies, to develop and propose an empirically grounded definition for imagination (research question three).

### 3.3 Analysis

In this integrated mixed method research synthesis, qualitative, quantitative, and mixed method studies were included in the final sample. Extracted data were quantized or qualitized according to the research question. For instance, qualitative methods were used to examine Research Question 1 (How is imagination defined as a construct in empirical studies related to creativity?) whereas quantitative methods were used to answer Research Question 1a (Do the authors (explicitly, implicitly, not at all) define imagination in the paper?). We also used thematic synthesis to coalesce and integrate the findings from studies included in this review.

Thematic analysis was used to identify and interpret emerging trends, patterns, and correlates in this review (Braun and Clarke, 2006). Thematic analysis is a flexible approach and lends itself to inductive and deductive coding approaches (Braun and Clarke, 2006; Miles and Huberman, 1994; Naeem et al., 2023). In this review, familiarity with the data was accomplished during the review of identified articles. Key passages and data were marked and labeled within each article stored in Zotero. We used this to generate initial codes and develop a coding guide. In addition to the initial codes generated from our research questions (e.g., definition of imagination, imagination in the creative process), extracted data included study design, geographical location, sample characteristics (e.g., sample size, age group, discipline), study purpose, measure(s)/instrument(s) used, variables, and key findings. Articles were independently reviewed and coded by both reviewers. Intercoder agreement was assessed with an initial agreement rate of 90%. Disagreements were discussed until consensus was reached. All four accounts in which disagreement occurred were related to vague or ambiguous definitions noted within the article by both reviewers. During the data extraction phase, both reviewers noted and selected keywords commonly used within the articles (Naeem et al., 2023), such as keywords that appeared in imagination definitions. After data extraction and initial coding, both reviewers searched for, reviewed, and defined themes. In general, we adhered to themes that were identified by both reviewers. Intercoder agreement was 86% initially; reviewers discussed any disagreements until consensus was achieved.

## 4 Results

Publication dates for articles reviewed in this synthesis ranged from 2012 to 2024, with most of the studies coming from 2021 (*n* = 7). Most of the articles included in this synthesis were quantitative (*n* = 30), followed by 10 qualitative studies, one study using both quantitative and qualitative methods (no mixing), and one mixed method study. Just over half of the quantitative studies in this sample were correlational studies (*n* = 16), but there was a good mix of experimental (*n* = 7) and instrument development (*n* = 7) studies represented also. Qualitative studies included focus groups, interviews, observations, and case studies. Just over a quarter of the studies in our sample were conducted in Taiwan (*n* = 11), but our sample also included studies conducted in China (*n* = 6), Poland (*n* = 4), the United Kingdom (*n* = 4), Turkey (*n* = 3), the United States (*n* = 2), Mexico (*n* = 2), Indonesia (*n* = 2), and other countries, including Croatia, Egypt, Ethiopia, Malaysia, Norway, Serbia, and Sweden. Sample characteristics in the reviewed studies were typically convenient samples of university students across a variety of disciplines but also included samples of students in primary and secondary grade levels, parents, teachers, and a mix of adults (e.g., hotel employees, right-handedness). See Table 2 for sample characteristics. When examining the data for patterns related to definitions of imagination and its relationship to creativity, patterns were observed and themes emerged. Results are categorized and reported by research question.

**Table 2:** Sample characteristics (*N* = 42).

Article	Method	Participants	Location
Ahmed, A. (2021). Using the imaginative approach to develop university students' creative writing skills and efficacy. *J. Facul. Educ*. 32, 157–210. Available online at: https://jfeb.journals.ekb.eg/article_239605.html	QUAN	Undergraduate students	Egypt
Ayalew, Y., and Areaya, S. (2019). A positional map of mathematical imagination and creativity. *Philos. Math. Educ. J*. 35, 1–15. Available online at: https://www.exeter.ac.uk/research/groups/education/pmej/pome35/index.html	QUAL	High school students	Ethiopia
Bačlija Sušić, B., and Brebrić, V. (2024). Encouraging and assessing preschool children's musical creativity. *Early Years* 44, 328–340. doi: 10.1080/09575146.2022.2139356	QUAL	Kindergarten students	Croatia
Bauer, R. H., and Gilpin, A. T. (2023). Creativity in early childhood: how do imagination and self-regulation predict creativity in a story-stem task? *Psychol. Aesthet. Creat. Arts*. Advance online publication. doi: 10.1037/aca0000620	QUAN	Pre-school students	United States
Chen, H.-H., and Yuan, Y.-H. (2021). The study of the relationships of teacher's creative teaching, imagination, and principal's visionary leadership. *Sage Open* 11, 1–15. doi: 10.1177/21582440211029932	QUAN	Elementary school teachers	Taiwan
Cheong, C. M., Zhang, J., Yao, Y., and Zhu, X. (2022). The role of gender differences in the effect of ideal L2 writing self and imagination on continuation writing task performance. *Think. Skills Creat*. 46: 101129. doi: 10.1016/j.tsc.2022.101129	QUAN	High school students	China
Craft, A., Cremin, T., Burnard, P., Dragovic, T., and Chappell, K. (2013). Possibility thinking: culminative studies of an evidence-based concept driving creativity? *Educ. 3-13* 41, 538–556. doi: 10.1080/03004279.2012.656671	QUAL	Children 9–11 years old; primary school	United Kingdom
Dereli, F. (2023). Investigation of relationship between creativity potential and scientific imagination of gifted children and comparing them with their peers. *J. Gift Educ. Creat*. 10, 157–175. https://izlik.org/JA42MR42PB	QUAN	Children 5–7 years old	Turkey
Dziedziewicz, D., Oledzka, D., and Karwowski, M. (2013). Developing 4- to 6-year-old children's figural creativity using a doodle-book program. *Think. Skills Creat*. 9, 85–95. doi: 10.1016/j.tsc.2012.09.004	QUAN	Children 4–6 years old	Poland
Eyada, L. D. (2022). Imagination and creativity in the work of middle school students. *J. Positiv. School Psychol*. 6, 1934–1942. https://journalppw.com/index.php/jpsp/article/view/7491	QUAL	Middle school students	Iraq
Frith, E., Gerver, C. R., Benedek, M., Christensen, A. P., and Beaty, R. E. (2022). Neural representations of conceptual fixation during creative imagination. *Creat. Res. J*. 34, 106–122. doi: 10.1080/10400419.2021.2008699	QUAN	Right-handed adults	United States
Fung, W. K., and Chung, K. K. H. (2022). Overexcitabilities and creative potential in the kindergarten context: the mediating role of children's playfulness. *Think. Skills Creat*. 46:101197. doi: 10.1016/j.tsc.2022.101197	QUAN	Parents and teachers of kindgarten students	China
Fung, W. K., Chung, K. K. H., and He, M. W. (2021). Association between children's imaginational overexcitability and parent-reported creative potential: cognitive and affective play processes as potential mediators. *J. Creat. Behav*. 55, 962–969. doi: 10.1002/jocb.501	QUAN	Parents of kindergarten students	China
Gonzalez Garcia, J., and Mukhopadhyay, T. P. (2019). The role and efficacy of creative imagination in concept formation: a study of variables for children in primary school. *Educ. Sci*. 9:175. doi: 10.3390/educsci9030175	QUAN/QUAL	Fifth grade students	Mexico
Grobelna, A. (2015). Role ambiguity: a problem or a challenge facing contemporary hospitality industry. The critical role of employees' creativity. *Int. J. Contemp. Manage* 14, 77–98. doi: 10.4467/24498939IJCM.15.005.4307	QUAN	Hotel employees	Poland
He, W. J., Wong, W. C., and Chan, M. K. (2017). Overexcitabilities as important psychological attributes of creativity: a Dabrowskian perspective. *Think. Skills Creativ*. 25, 27–35. doi: 10.1016/j.tsc.2017.06.006	QUAN	Students (grades 7–11)	China
Horng, R. Y., Wang, C. W., Yen, Y. C., Lu, C. Y., and Li, C. T. (2021). A behavioural measure of imagination based on conceptual combination theory. *Creativ. Res. J*. 33, 376–387. doi: 10.1080/10400419.2021.1943136	QUAN	University students	Taiwan
Hsu, Y. (2019). Advanced understanding of imagination as the mediator between five-factor model and creativity. *J. Psychol*. 153, 307–326. doi: 10.1080/00223980.2018.1521365	QUAN	University students	Taiwan
Hsu, Y., Chang, C. C., and Liang, C. (2015). The effects of creative personality and psychological influences on imagination. *Innovat. Educ. Teach. Int*. 52, 587–598. doi: 10.1080/14703297.2013.808404	QUAN	University students	Taiwan
Hsu, Y., Peng, L. P., Wang, J. H., and Liang, C. (2014). Revising the imaginative capability and creative capability scales: testing the relationship between imagination and creativity among agriculture students. *Int. J. Learn. Teach. Educat. Res*. 6, 57–70. http://ijlter.org/index.php/ijlter/article/view/118	QUAN	University students	Taiwan
Jankowska, D. M., Gajda, A., and Karwowski, M. (2019). How children's creative visual imagination and creative thinking relate to their representation of space. *Int. J. Sci. Educ*., 41, 1096–1117. doi: 10.1080/09500693.2019.1594441	QUAN	Preschool children (5 years old)	Poland
Jankowska, D. M., and Karwowski, M. (2015). Measuring creative imagery abilities. *Front. Psychol*. 6:Article 1591. doi: 10.3389/fpsyg.2015.01591	MM	Participants (5–55 years old)	Poland
Joseph, C., Tan, C. K., and Ambotang, A. S. (2023). Teachers' perceptions on brainstorming and imagination techniques in enhancing creativity in drawing among preschool students. *Int. J. Modern Educ*. 5, 24–36. Available online at: https://gaexcellence.com/ijmoe/article/view/1836	QUAL	Preschool teachers	Malaysia
Kaya, F., and Acar, S. (2019). The impact of originality instructions on cognitive strategy use in divergent thinking. *Think. Skills Creat*. 33: Article 100581. doi: 10.1016/j.tsc.2019.100581	QUAN	High school students	Turkey
Kusmaryono, I., and Maharani, H. R. (2021). Imagination and creative thinking skills of elementary school students in learning mathematics: a reflection of realistic mathematics education. *Element. Islam. Teach. J*. 9, 287–308. doi: 10.21043/elementary.v9i2.11781	QUAL	Sixth grade students and one classroom teacher	Indonesia
Liang, C., and Lin, W.-S. (2015). The interplay of creativity, imagination, personality traits, and academic performance. *Imag. Cognit. Personal*. 34, 270–290. doi: 10.1177/0276236614568638	QUAN	University students	Taiwan
Lin, W.-S., Hsu, Y., and Liang, C. (2014). The mediator effects of conceiving imagination on academic performance of design students. *Int. J. Technol. Des. Educ*. 24, 73–89. doi: 10.1007/s10798-013-9244-x	QUAN	University students (undergraduate and graduate)	Taiwan
Lin, H.-H., and Tsau, S.-Y. (2013). The development of an imaginative thinking scale. *Imag. Cognit. Pers*. 32, 207–238. doi: 10.2190/IC.32.3.b	QUAN	First-year undergraduates	Taiwan
Liu, H.-Y., Chang, C.-C., Wang, I.-T., and Chao, S.-Y. (2020). The association between creativity, creative components of personality, and innovation among Taiwanese nursing students. *Think. Skills Creat*. 35:Article 100629. doi: 10.1016/j.tsc.2020.100629	QUAN	University students	Taiwan
Ness, I. J., and Dysthe, O. (2020). Polyphonic imagination: Understanding idea generation in multidisciplinary groups as a multi-voiced stimulation of fantasy. *Creat. Res. J*. 32, 30–40. doi: 10.1080/10400419.2020.1712163	QUAL	People with varied educational and experiential backgrounds	Norway
Puente-Díaz, R., and Cavazos-Arroyo, J. (2017). Creative self-efficacy: the influence of affective states and social persuasion as antecedents and imagination and divergent thinking as consequences. *Creat. Res. J*. 29, 304–312. doi: 10.1080/10400419.2017.1360067	QUAN	College students	Mexico
Richard, V., Aubertin, P., Yang, Y. Y., and Kriellaars, D. (2020). Factor structure of play creativity: a new instrument to assess movement creativity. *Creativ. Res. J*. 32, 383–393. doi: 10.1080/10400419.2020.1821567	QUAN	Children (grades 4–6)	Not specified
Siswanto, I., Wu, M., and Ko, C. (2020). The impact of imagination and affective creativity on university students' creative thinking: a verification through structural equation model. *Int. J. Innovat. Creat. Change*, 13, 371–389. Available online at: https://www.ijicc.net/index.php/volume-13-2020/197-vol-13-iss-12	QUAN	University students	Taiwan
Smees, R., Simner, J., and Rinaldi, L. J. (2024). Are children with autistic traits more or less creative? Links between autistic traits and creative attributes in children. *J. Creat. Behav*. 58(3), 328–341. doi: 10.1002/jocb.650	QUAN	Children and parents	United Kingdom
Sungurtekin, S. (2021). Classroom and music teachers perceptions about the development of imagination and creativity in primary music education. *J. Pedagog. Res*. 5(3), 164–186. doi: 10.33902/JPR.2021371364	QUAL	Teachers	Turkey
Taylor Bunce, L., and Boerger, E. A. (2022). Openness to experience mediates the relation between fantasy proneness and creative thinking. *Imaginat. Cognit. Personal*. 42, 192–214. doi: 10.1177/02762366221120214	QUAN	Undergraduate students	United Kingdom
von Stumm, S., and Scott, H. (2019). Imagination links with schizotypal beliefs, not with creativity or learning. *Br. J. Psychol*. 110, 707–726. doi: 10.1111/bjop.12369	QUAN	Undergraduate students and adult volunteers	United Kingdom
Winarningsih, U., Khasanah, U., and Fithayanti, W. (2024). The implementation of loose parts play in enhancing creativity and fostering children's imagination at RA Al Hidayah Plelen in 2024. *J. Profesionalisma Guru* 1, 378–382. https://journal.maalahliyah.sch.id/index.php/jpg/article/view/265	QUAL	Students and teachers	Indonesia
Wu, M., Siswanto, I., and Ko, C. (2017). The influential factors and hierarchical structure of college students' creative capacities—an empirical study in Taiwan. *Think. Skills Creat*. 26, 176–185. doi: 10.1016/j.tsc.2017.10.006	QUAN	University students	Taiwan
Zhang, M., Liu, G., Liu, W., Zhou, Z., Chen, S., Liang, Z., and Zhao, Q. (2024). How we imagine poetic constructions: The role of episodic simulation in poetry composition. *Creat. Res. J*. Advance online publication. doi: 10.1080/10400419.2024.2443607	QUAN	University students	China
Zheng, Y., and Leung, B.-W. (2021). Cultivating music students' creativity in piano performance: a multiple-case study in China. *Music Educ. Res*. 23, 594–608. doi: 10.1080/14613808.2021.1977787	QUAL	University students	China
Zivkovic, Z., Nikolic, S. T., Doroslovacki, R., Lalic, B., Stankovic, J., and Zivkovic, T. (2015). Fostering creativity by a specially designed Doris tool. *Think. Skills Creat*. 17, 132–148. doi: 10.1016/j.tsc.2015.06.004	QUAN	High school students (15–18 years old)	Serbia, Hungary, Italy, Austria, Croatia

### 4.1 Defining imagination

First, we analyzed the data to determine whether imagination was defined in each article. Across all studies in this sample, 23 explicitly defined imagination, 14 implicitly defined it (using descriptive terms or phrases), and five did not define imagination at all. Definitions were classified as explicit when authors clearly constructed and stated a definition or adopted a definition from existing literature to guide their study. For example, Hsu et al. (2014) explicitly stated in their article, “In this study, imagination refers to the capability of students to initiate, conceive, and transform their ideas into schoolwork and/or perform related actions” (p. 59). Intercoder agreement for coding definitions was high, reflecting good agreement (93%). All three discrepancies in this area related to vague descriptions of imagination, such as instances where authors framed imagination within another construct (e.g., Joseph et al., 2023) or made associations with creativity without distinguishing between the two (e.g., Bačlija Sušić and Brebrić, 2024; Puente-Díaz and Cavazos-Arroyo, 2017; Winarningsih et al., 2024; Zheng and Leung, 2021).

Reviewers also coded definitions for frequency of keywords used. In over half the definitions provided by authors, imagination was defined or described as an ability (*f* = 20), and the most frequently used words across explicit and implicit definitions were mental (*f* = 14; e.g., Ahmed, 2021; Frith et al., 2022; von Stumm and Scott, 2019), image(s) (*f* = 14; e.g., Hsu, 2019; Wu et al., 2017), idea(s) (*f* = 14; e.g., Chen and Yuan, 2021; Cheong et al., 2022; Kaya and Acar, 2019), experience (*f* = 13; e.g., Jankowska et al., 2019; Siswanto et al., 2020; Zivkovic et al., 2015), and new (*f* = 11; e.g., Lin and Tsau, 2013; Ness and Dysthe, 2020; Sungurtekin, 2021). Some authors attributed imagination to a way of thinking (*f* = 8), such as magical thinking (Fung et al., 2021; He et al., 2017), possibility thinking (Craft et al., 2013; Zivkovic et al., 2015), metaphorical thinking (Hsu, 2019), and wishful thinking (Kusmaryono and Maharani, 2021). Although used in 13 definitions, the keyword transform appeared only in explicit definitions (e.g., Dziedziewicz et al., 2013; Eyada, 2022; Jankowska and Karwowski, 2015). Explicit and implicit definitions associated with each article are presented in Table 3.

**Table 3:** Explicit and implicit definitions of imagination.

Authors	Explicit definition	Implicit definition
Ahmed, 2021	“As a result, imaginative learning is viewed as a mental talent to innovate. Imaginative thinking is defined by Merriam-Webster (n.d.) as the ability to generate a mental image of something that is not present to the senses or has never been fully observed in actuality” (p. 185).	
Ayalew and Areaya, 2019	“We also viewed imagination as it involves aspects of thinking, mental picture and spatial representations.” (p. 3); "The concept imagination is an abstract and philosophical. Thus, it is a qualitative construct; and so, indirect method of investigation would be an essential approach. There are nine recognized indicators to assess human imagination: transformation, crystallization, effectiveness, elaboration, exploration, intuition, novelty, productivity, and sensibility (Liang et al., 2012). The last six indicators would be labeled into creative imagination. That means, most aspects of imagination are creative, and some are not” (p. 5).	
Bauer and Gilpin, 2023		“Imaginative children are more likely than peers to have a proclivity for fantastical play, toys, and games, have imaginary friends, impersonate imaginary characters, and believe in fantastical entities (Pierucci et al., 2014). Their imaginative thinking and behaviors may be especially generative due to a lack of constraint to real-life possibilities. Because of this preference, imaginative children may spend more time engaging in a generative process and this may generalize outside the play realm and into the generative process in creativity” (p. 2).
Chen and Yuan, 2021	“Thus, in this study, the researchers defined imagination as a mental ability that can transcend spatial and temporal limitations to form images. The ability is based on the combination of an individual's experience. This mental ability integrates the perceptual ability to visualize dynamic process, such as processing, transformation, reorganization, and mental innovation. Imagination enables an individual to have new ideas on things that they have never experienced, where these ideas are reflected in an individual's work, life, and plans for the future” (p. 2).	
Cheong et al., 2022	“Imagination is defined as the cognitive ability to envisage the world beyond current epistemic modality (Castoriadis and Curtis, 1997). It is the mental process of bridging images and ideas together, re-structuring for a novel purpose (He and Shum, 2005)” (p. 3).	
Craft et al., 2013		“Being imaginative… identified as the core component of Possibility Thinking (PT)” (p. 9); “Being imaginative: ‘as if' thinking” (p. 17).
Dereli, 2023	“Etymologically, the word imagination derives from the Latin word “imago” meaning image or mental representation… The act of imagining is often used in the sense of visualizing images. Imagination therefore means visualizing or picturing something in the mind (Jagla, 1994). In the dictionary of the Turkish Language Association, ‘imagination' is defined as ‘the ability of the mind to create imagination, imagination, imagery, fancy; the power to establish a connection between the elements of past experiences and present experiences; the ability to design an object without the object being in front of us' [Türk Dil Kurumu (TDK), n.d.]” (p. 158).	
Dziedziewicz et al., 2013	“Creative imagination is the ability to transform available and remembered data into new and original mental images” (p. 86).	
Eyada, 2022	“Imaginative processes transform memory from images received through the senses in an analytical and synthesis manner to achieve its new imaginative formulation” (p. 1934); “The ability to shape images of objects – the scenes of existence, the imagination preserves the perceptions of common sensory and images of sensors... Imagination is associated with sense, cognition, and remembrance. While imagining it, an individual selects, arranges, and mutates to realities that could not be realized through the senses” (p. 1935).	
Frith et al., 2022		“In the present research, we experimentally manipulated associative conceptual constraints to explore whether fixation on salient mental representations of visual stimuli impacted neural pattern similarity during divergent creative imagination (i.e., imagining novel labels for ambiguous line drawings; Jankowska and Karwowski, 2015)” (p. 106).
Fung and Chung, 2022		“Overexcitabilities reflect one's inherited tendencies to be exceptionally aroused by stimulations (Dabrowski, 1964). Dabrowski (1964) proposed five forms of overexcitability including imaginational (lively imaginations and extensive associations)…” (p. 2).
Fung et al., 2021		“Dabrowski (1964) identified five forms of over-excitability… imaginational (vivid imaginations and rich associations)… Children with heightened imaginational over-excitability have an inherent tendency to be ‘super stimulated' by internal and external stimuli such as fantasies, images, metaphors, and personification and they tend to express their exceptional arousal through vivid imagination, magical thinking, and dramatization” (p. 963).
Gonzalez Garcia and Mukhopadhyay, 2019	“In this paper, we shall use the term ‘creative imagination' to denote integrated neurobehavioral functions that employ imagination” (p. 1); “Imagination is a ‘new psychological process' for the child. It represents a ‘specifically human form of conscious activity.' Like all functions of knowledge, it arises originally from ‘action.' Since creative imagination can be discovered in the products of creative life, especially of children, we tried to see how elements of representation, by storytelling or drawing, are incorporated in meaningful representations” (p. 2).	
Grobelna, 2015		“Individuals of high openness to experience are characterized by intellectual curiosity; they are creative and have vivid imagination (Costa and McCrae, 1998)… Based on the above discussion, it can be assumed that persons who have vivid imagination, plenty of good ideas, and who understand things quickly may be more creative than those individuals who cannot be described in this way; therefore, the personality variable defined as intellect/imagination may have a relationship with employees' creativity” (p. 84).
He et al., 2017		“It has been suggested that a person with imaginational OE [Overexcitabilities] is passionate about imagination, fantasies, dreams, dramatizations, inventions, detailed visualizations, associative thinking, and magical thinking. Imaginational OE is also associated with a desire for novelty, variety, and the unusual” (p. 28).
Horng et al., 2021	“In this study, imagination is proposed to be a mental activity involving conceptual combination” (p. 376); Imagination is defined as the ability to combine two or more unrelated concepts to produce an original concept” (p. 379).	
Hsu, 2019	“Imagination is inner mental activity and refers to a wide range of activities such mental imagination, metaphorical thinking, nonverbal communication, and internal visualization (Egan, 2007; Eisner, 2002). Scholars have also noticed that capability-oriented imagination—a mental power distinguished from fantasy that can be measured and may demonstrate different characteristics in the process of altering mental images of what is or could be—enhances creativity (e.g., Liang and Chia, 2014; Stokes, 2014; Zittoun and Cerchia, 2013)” (p. 308).	
Hsu et al., 2015	“In this study, imagination refers to the process of transforming the inner imagery of film students, when they face a film production task” (p. 588).	
Hsu et al., 2014	“Imagination is defined as ‘a power of the mind' (Perdue, 2003) that enables people to transcend experience and construct alternative possibilities to organize fragmented situations into meaningful and complete concepts (Passmore, 1985)… In this study, imagination refers to the capability of students to initiate, conceive, and transform their ideas into schoolwork and/or perform related actions” (p. 59).	
Jankowska et al., 2019	“Creative visual imagination (the ability to imagine something that we have never previously experienced)” (p. 1097); In this study, we use the definition of children's creative visual imagination put forward in the recent conjunctional model of creative imaging ability (CMCIA; Dziedziewicz and Karwowski, 2015). The CMCIA posits that, creative visual imagination has three interrelated dimensions: vividness – the ability to create expressive and highly complex images; originality – the ability to produce unique images, based on past observations but transcending them is some significant way; and transformative ability – the ability to transform generated images” (p. 1099).	
Jankowska and Karwowski, 2015	“The activity of visual imagination encompasses creating, interpreting, and transforming vivid mental representations…In this model, creative imagination is defined as ability to create and transform representations that are based on the material of past observations but that significantly transcend them—by creating the so-called creative representations” (p. 1).	
Kaya and Acar, 2019		“People can generate ideas beyond their experiences through using their imagination and other cognitive strategies” (p. 3).
Kusmaryono and Maharani, 2021	“Imagination is a work of the mind in developing a broader thought than what has been seen, heard, and felt (Pelaprat and Cole, 2011; Siswono, 2011). Imagination is the power of thought to imagine (in wishful thinking) or create images (paintings, essays, etc.) of events based on reality or one's general experience (Pelaprat and Cole, 2011; Venturi, 2015)” (p. 290).	
Liang and Lin, 2015	“Initiating imagination refers to the capability to explore the unknown and productively originate novel ideas (Beaney, 2005; Liu and Noppe-Brandon, 2009). Conceiving imagination refers to the capability to mentally grasp the core of a phenomenon utilizing personal intuition and sensibility, and the capability to formulate effective ideas for achieving a goal through concentration and logical dialectics (Cartwright and Noone, 2006; Kunzendorf, 1982; Reichling, 1990). Transforming imagination refers to the capability to crystallize abstract ideas and reproduce what is known across different domains and in various situations (Kunzendorf, 1982; Liu and Noppe-Brandon, 2009; Perdue, 2003). Imagination for designers is about seeing things in a new light and being able to achieve different results” (p. 272).	
Lin et al., 2014	“In the current study, ‘imagination' thus refers to the capability of design students to initiate, conceive and transform their ideas into design plans and/or related actions” (p. 75).	
Lin and Tsau, 2013	“‘Imagination' is referred to as a transforming, creative activity from the concrete toward a new concrete with the help of abstraction (Moran and John-Steiner, 2003, p. 13)” (pp. 208–209); “the ‘Imaginative Thinking Scale,' which we will present in this article, is based on the specific definition of imagination, involving initiation, fluency, flexibility, and originality” (p. 217).	
Liu et al., 2020		“In their view, ‘Creative thinking enables students to apply their imagination to generating ideas, questions, and hypotheses, experimenting with alternatives and to evaluating their own and their peers' ideas, final products, and processes” (p. 2).
Ness and Dysthe, 2020	“Imagination is, according to Vygotsky (2004), the process whereby the mind takes up known elements and uses and combines them in new ways. An individual's capacity to make connections between objects, events, and tools in his or her life is directly defined by how much that person can imagine someone else's experiences. Imagination, ‘becomes the means by which a person's experience is broadened, because he has to imagine what he has not seen, can conceptualize something from another person's narration and description of what he himself has never directly experienced'… based on the above, this paper conceptualizes imagination as a higher mental function, mediated by psychological tools (for instance the way the group members used language and terminology in their communication) but also other technological tools used to help them convey their meaning to the others” (p. 32).	
Richard et al., 2020		“Creative imagination emphasizes on the attributes of initiation and originality (Colello, 2007). Because creative imagination involves novelty, it may partly explain why it was highly correlated to originality which also encompasses this criterion” (p. 390).
Siswanto et al., 2020	“Lin et al. (2014) define imagination as individual's ability to think of something unreal and initiate, conceive, and change their ideas into intention plans and actions… Imagination allows the individual to go beyond real experiences and build some alternative possibilities into a new meaning (Liang et al., 2013). Hence, it can be concluded that imagination is individual ability to transfer their previous feeling retrospectively, thought, and experiences to conceptually visualise something that might be does not exist yet in the form of new products, process, or events” (p. 374).	
Smees et al., 2024		“[Autism Spectrum Quotient] AQ-Imagination largely involves the child's ability to engage with fictional worlds (e.g., through fiction), while AQ-Attention to detail measures the capacity to see details others miss” (p. 329).
Sungurtekin, 2021		“Creative thinking is the ability to think imaginatively and ‘the act of manipulating old ideas and creating new and original musical ideas' (as cited in Reesman, 2015, p. 11)” (p. 166).
Taylor Bunce and Boerger, 2022		“This trait [Openness to Experience] includes Aesthetics (artistic interests), Feelings (emotionality), Actions (adventurousness), Ideas (intellectual curiosity), Fantasy (imagination), and Values (psychological liberalism)” (p. 196).
von Stumm and Scott, 2019	“Imagination refers to creating mental representations of concepts, ideas, and sensations that are not contemporaneously perceived by the senses” (abstract); “Imagination can be broadly defined as the tendency to create ‘mental representations of concepts, ideas, and sensations in the mind that are not contemporaneously perceived by the senses [and ranges] from the recreation of images or sensory perceptions in the mind that were previously seen or experienced in reality [...] to crafting images anew independent of prior actual sensory input [...]' (Scott and von Stumm, 2017, p. 1)” (p. 707).	
Wu et al., 2017	“The term imagination originates from the Latin verb imaginari, meaning ‘to picture oneself.' Imagination is an action to form new ideas, images or concepts of external objects that only exist or occur in an individual mind (Merriam-Webster, n.d.; Oxford Dictionaries, 2016). Furthermore, some scholars also define imagination as the ability to think of what is not present, unreal or even absurd, and it appears to give people almost unlimited conceptual powers (Beaney, 2005; Liang et al., 2012). Similarly, imagination	
	refers to the capability of students to think of something that is not present/does not exist and initiate, conceive, and transform their ideas into design plans and/or related actions” (p. 178).	
Zhang et al., 2024		“Episodic simulation is the ability to flexibly recombine episodic details to imagine scenes or events” (p. 1).
Zivkovic et al., 2015	“The general definition of imagination is: ‘Imagination is the ability to think of all things as possible' (Kangas, 2010, 2). The more comprehensive explanation sees ‘imagination as an aspect of reflective thinking that enables us to create ideas that not only go beyond what is given but are effective, in the sense that they are likely to transform experience as intended… Imagination is the internal imagery of a creator whereas creativity and creations are the outward manifestation of imagination' (Liang et al., 2013, 110)” (p. 133).	

This table does not include authors who did not define imagination (*n* = 5).

#### 4.1.1 Characteristics of and slight distinctions between explicit and implicit definitions

Although imagination was defined both explicitly and implicitly, these approaches differed in their level of precision, clarity, and connection to the study's context. Overall, explicit definitions were generally clearer, more study-specific, and often connected to a particular domain (e.g., math, science, writing) or to the instrument used to measure the construct. In other words, authors who explicitly defined imagination were clearer about what imagination meant and what it meant with regard to the context of their study. Although both explicit and implicit definitions were informed by empirical literature, implicit definitions often used existing literature, particularly literature on related constructs (e.g., creativity, ways of thinking, overexcitabilities), to paint a picture of sorts of imagination. Implicit definitions allowed meaning to emerge through associated characteristics and constructs rather than a formal definition. Despite these distinctions, explicit and implicit definitions suggested a shared understanding of imagination across the studies in our sample.

Authors who explicitly defined imagination tended to use direct, study-specific language and were more likely to specify the domain of interest and/or their measurement approach. In many articles, authors used direct statements, such as “we use the definition of…” (Jankowska et al., 2019, p. 1099) or “we viewed imagination as…” (Ayalew and Areaya, 2019, p. 3), to explicitly define imagination in terms of what the construct meant “in [their] study or paper” (e.g., Chen and Yuan, 2021; Gonzalez Garcia and Mukhopadhyay, 2019, p. 1; Hsu et al., 2015; Jankowska and Karwowski, 2015; Lin et al., 2014). Others similarly used direct language while also connecting imagination to a specific domain, such as art, film, and design (e.g., Gonzalez Garcia and Mukhopadhyay, 2019; Hsu et al., 2015; Liang and Lin, 2015). One author explicitly defined both imagination and scientific imagination (Dereli, 2023). In contrast, Ayalew and Areaya (2019) used the term mathematical imagination in the title of their paper but did not provide a definition for the term, although they did explicitly define imagination, more generally.

Alignment with the instrument used in the study was also more prevalent in studies that explicitly defined imagination. For example, Horng et al. (2021) created and tested an instrument (two parallel forms of an imagination test that included items of unrelated noun-pairs) that strongly aligned with their definition of imagination: “a mental activity involving conceptual combination” (p. 376). For others, mental imagery was a strong component of how they defined imagination in which authors used performance tasks to examine the construct, such as visualization (Ahmed, 2021), drawing (Eyada, 2022), and writing (Cheong et al., 2022). In some instances, this alignment between authors' definitions and the instruments used in their studies inherently reflected underlying factor structures of imagination. Lin and Tsau (2013) developed and used the Imaginative Thinking Scale (four factors) which was “based on the specific definition of imagination, involving initiation, fluency, flexibility, and originality” (p. 217). Lin et al. (2014) conducted a study with university students enrolled in design, stating “In the current study, ‘imagination' thus refers to the capability of design students to initiate, conceive, and transform their ideas into design plans and/or related actions” (p. 75). This definition was strongly linked to the instrument used to measure the construct: Imaginative Capability Scale (ICS). The ICS is composed of three factors: (a) initiating, or trying something unfamiliar or generating new ideas; (b) conceiving, or using one's personal intuition to comprehend phenomena, and generate effective ideas for achieving one's goals; and (c) transforming, or reconstructing mental images from less precise memories that span across various domains and situations (Liang and Chia, 2014). In this review, five studies used the ICS, adopting the three-factor model of imagination (e.g., Hsu, 2019; Hsu et al., 2015, 2014; Liang and Lin, 2015; Lin et al., 2014); however, all five were conducted by the same author multiple times or seemingly affiliated author groups in Taiwan. Both studies by Jankowska and colleagues used the Test of Creative Imagery Abilities (TCIA) to measure creative visual imagination, or the ability to imagine something never experienced (Jankowska et al., 2019; Jankowska and Karwowski, 2015). The TCIA measures three components of creative imagination (vividness, originality, transformation) as they relate to visual imagery. Overall, alignment between explicit definitions and measurement approaches strengthened conceptual clarity and consistency in how imagination was operationalized.

Authors who implicitly defined imagination often leaned more on literature-based descriptions rather than clearly connecting their study to a formal definition or guiding framework. Implicit definitions typically followed two general approaches: (a) situating imagination within the context of another construct (e.g., overexcitabilities, Fung and Chung, 2022; openness to experience, Taylor Bunce and Boerger, 2022); or (b) describing imagination through related behaviors (e.g., possibility thinking, Craft et al., 2013; fantasy and imaginary play, Bauer and Gilpin, 2023). In short, authors implicitly defined imagination by describing the construct without directly saying what it is. Despite this, researchers' implicit definitions revealed common patterns and characteristics associated with imagination.

When authors used other constructs to describe imagination, there were still enough related descriptors to convey an implicit meaning of the construct. For example, Fung et al. (2021) drew on the literature on overexcitabilities to describe imagination: “Dabrowski (1964) identified five forms of over-excitability… imaginational (vivid imaginations and rich associations)…” (p. 963). Similarly, Grobelna (2015) and Taylor Bunce and Boerger (2022) conceptualized imagination as a personality variable—intellect/imagination and openness to experience, respectively. Smees et al. (2024) framed imagination within traits associated with autism, noting that “[Autism Spectrum Quotient] AQ-Imagination largely involves the child's ability to engage with fictional worlds (e.g., through fiction)” (p. 329). These examples also parallel patterns observed in explicit definitions: when imagination was situated within constructs such as personality, overexcitability, and autism, the implicit definitions aligned with corresponding subscales of the instruments used in the study (e.g., Adolescent AQ, Smees et al., 2024; International Personality Item Pool, Grobelna, 2015; Taylor Bunce and Boerger, 2022; Overexcitability Questionnaire-Two, Fung and Chung, 2022; Fung et al., 2021; He et al., 2017).

Authors who defined imagination through related behaviors most often emphasized behaviors associated with creativity, such as generating and manipulating ideas (e.g., Kaya and Acar, 2019; Liu et al., 2020; Sungurtekin, 2021) and novelty (Richard et al., 2020). Although Frith et al. (2022) included commonly used creativity descriptors (e.g., divergent, novel), their conceptualization of imagination placed greater emphasis on mental imagery. Bauer and Gilpin (2023), an outlier among implicit definitions, focused instead on characteristics of imaginative children, highlighting associations with fantasy play and imaginary characters. They also framed imaginative thinking as a generative process linked to creativity, a perspective echoed by Kaya and Acar (2019). Similarly, Craft et al. (2013) described “as if” thinking as a key feature of being imaginative (p. 12). In sum, when not explicitly defined, imagination was characterized through behaviors and processes—particularly those tied to creativity—rather than formally stating what imagination is.

Collectively, these patterns highlight a key distinction in how imagination was conceptualized across studies in our sample: either as a clearly defined, operationalized construct or as a set of inferred characteristics. Yet, both still point to a shared conceptual core. Several overlapping themes were also noted and are discussed in the following sections for both implicit and explicit definitions. See Table 4 for emergent themes across definitions of imagination, associated key words or phrases used for coding, and representative quotes.

**Table 4:** Emerging themes from definitions of imagination: key words/phrases and representative quotes.

Themes	Extracted key words or phrases	Example quote
Imagination involves mental representations and imagery	mental representations	“In the present research, we experimentally manipulated associative conceptual constraints to explore whether fixation on salient mental representations of visual stimuli impacted neural pattern similarity during divergent creative imagination (i.e., imagining novel labels for ambiguous line drawings; Jankowska and Karwowski, 2015)” (Frith et al., 2022, p. 106).
	process of transforming inner imagery	“In this study, imagination refers to the process of transforming the inner imagery of film students, when they face a film production task” (Hsu et al., 2015, p. 588).
	transform available and remembered data into new and original mental images	“Creative imagination is the ability to transform available and remembered data into new and original mental images” (Dziedziewicz et al., 2013, p. 86).
Imagination as the transformation and transcendence of prior experience	based on reality or one's general experience	“Imagination is a work of the mind in developing a broader thought than what has been seen, heard, and felt (Pelaprat and Cole, 2011; Siswono, 2011). Imagination is the power of thought to imagine (in wishful thinking) or create images (paintings, essays, etc.) of events based on reality or one's general experience (Pelaprat and Cole, 2011; Venturi, 2015)” (Kusmaryono and Maharani, 2021, p. 290).
	enables people to transcend experience	“Imagination is defined as—a power of the mind (Perdue, 2003) that enables people to transcend experience and construct alternative possibilities to organize fragmented situations into meaningful and complete concepts (Passmore, 1985)” (Hsu et al., 2014, p. 59).
	the means by which a person's experience is broadened, imagine what has not seen, conceptualize what has never directly experienced	“Imagination, ‘becomes the means by which a person's experience is broadened, because he has to imagine what he has not seen, can conceptualize something from another person's narration and description of what he himself has never directly experienced'…” (Ness and Dysthe, 2020, p. 32).
Imagination is the driving force behind new ideas	generate ideas beyond their experiences	“People can generate ideas beyond their experiences through using their imagination and other cognitive strategies” (Kaya and Acar, 2019, p. 3).
	initiate, conceive, and transform their ideas	“In the current study, ‘imagination' thus refers to the capability of design students to initiate, conceive and transform their ideas into design plans and/or related actions” (Lin et al., 2014, p. 75).
	ability to think of all things possible, reflective thinking, create ideas that go beyond but effective, symbolic expression of ideas	“The general definition of imagination is: ‘Imagination is the ability to think of all things as possible' (Kangas, 2010, p. 2). The more comprehensive explanation sees ‘imagination as an aspect of reflective thinking that enables us to create ideas that not only go beyond what is given but are effective, in the sense that they are likely to transform experience as intended… Imagination is an essential human capacity in various activities such as the pursuit of creativity and innovation, the symbolic expression of ideas, and critical thinking” (Zivkovic et al., 2015, p. 133).
Imagination is an essential piece of human cognition	cognitive ability, mental process	“Imagination is defined as the cognitive ability to envisage the world beyond current epistemic modality. It is the mental process of bridging images and ideas together, re-structuring for a novel purpose” (Cheong et al., 2022, p. 3).
	higher mental function	“Based on the above, this paper conceptualizes imagination as a higher mental function” (Ness and Dysthe, 2020, p. 32).
	mental process causing a new idea to emerge in the mind, mental activity involving conceptual combination	“Imagination refers to the mental process causing a new idea to emerge in the mind… In this study, imagination is proposed to be a mental activity involving conceptual combination” (Horng et al., 2021, p. 376).

Definitional aspects were coded under multiple themes as appropriate (e.g., The Cheong et al. (2022) definition is an example in which the definition supported all four themes).

#### 4.1.2 Imagination involves mental representations and imagery

Twenty of the studies in this review included mental representation, imagery, or visualizations in their definitions of imagination. For instance, imagination was described as involving “mental pictures and spatial representations” (Ayalew and Areaya, 2019, p. 5), a “mental process of bridging images and ideas together” (Cheong et al., 2022, p. 3), and “internal imagery” (Zivkovic et al., 2015, p. 133). Some authors elaborated on the role of imagery in imagination. For example, Jankowska et al. (2019) described imagination as encompassing complex and unique mental images, whereas others emphasized the ability to generate mental images not present to the senses (e.g., Ahmed, 2021) or conceptualized imagery in relation to imaginational overexcitability (e.g., Fung et al., 2021; He et al., 2017). Further, mental imagery or visualizations were often linked to the combination of memories or experiences, while still requiring an element of the unknown (e.g., Dereli, 2023; Hsu, 2019; Liang and Lin, 2015) or novelty (e.g., Dziedziewicz et al., 2013; von Stumm and Scott, 2019; Wu et al., 2017). In other words, imagination differed from general ideation in that ideas took the form of mental imagery and served as a foundation to solve seemingly unsolvable problems, or in some cases, to “visualize something that [may] not exist yet” (Siswanto et al., 2020, p. 374). Across this theme, authors consistently emphasized imagery, visualization, and representation as internal processes underlying imagination.

#### 4.1.3 Imagination as the transformation and transcendence of prior experience

In multiple studies (*n* = 20), authors conceptualized imagination as a transformative process often grounded in an individual's prior experiences, including memories (Eyada, 2022) or past observations (Jankowska and Karwowski, 2015), with some authors also emphasizing transcendence beyond experience (e.g., Cheong et al., 2022; Hsu et al., 2014; Jankowska et al., 2019). Nine articles explicitly referred to former experiences as a basis for one's mental imagery (e.g., Chen and Yuan, 2021; Dereli, 2023; Kusmaryono and Maharani, 2021; von Stumm and Scott, 2019) whereas others highlighted the transformation of memories, images, and ideas into something new in their definitions of imagination (e.g., Jankowska et al., 2019; Kusmaryono and Maharani, 2021; Siswanto et al., 2020; Sungurtekin, 2021). Some referred to imagination as future-oriented (Chen and Yuan, 2021; Lin and Tsau, 2013; Siswanto et al., 2020) where new constructions are informed by memories and experiences (Ahmed, 2021; Eyada, 2022) and experiences are reshaped through the imagination (Ness and Dysthe, 2020; Zivkovic et al., 2015). Overall, these studies pointed to imagination as a process that draws on memories and everyday experiences to generate ideas for the future, including both problem-solving and novel possibilities.

#### 4.1.4 Imagination is the driving force behind new ideas

Of the 42 articles examined, 18 articles used new understandings, innovative ideas, and new connections as defining characteristics of imagination. The creation of novel ideas through imagination enables individuals to move beyond habitual understandings and perceive things in a “new light” (Liang and Lin, 2015, p. 272). Further, Zivkovic et al. (2015) characterized imagination as the ability to generate new ideas that transcend our current experience. Others described imagination as combining elements in new ways (Ness and Dysthe, 2020) or bringing images and ideas together in a novel way (Cheong et al., 2022) and building new meanings (Siswanto et al., 2020) while some authors linked imagination to the propensity for the unusual (Taylor Bunce and Boerger, 2022; von Stumm and Scott, 2019). Sungurtekin (2021) characterized imagination within the context of music and as an act of forming old ideas into new ideas whereas Grobelna (2015) mentioned “plenty of good ideas” (p. 84) in their explanation of imagination. Kaya and Acar (2019) focused their description of imagination on generating ideas beyond one's experiences. Less commonly, authors included possibility thinking (Craft et al., 2013; Hsu et al., 2014; Siswanto et al., 2020; Zivkovic et al., 2015) and associative thinking (Fung and Chung, 2022; Fung et al., 2021; He et al., 2017). Craft et al. (2013) defined possibility thinking as what if thinking, relating it to the “seed of imagination” (p. 7) whereas some linked possibility thinking to the incubation phase (Liang and Lin, 2015; Lin et al., 2014). Notably, references to associative thinking were minimal and primarily appeared within definitions of imaginational overexcitability, often involving overlapping author groups (e.g., Fung and Chung, 2022; Fung et al., 2021; He et al., 2017).

#### 4.1.5 Imagination is an essential piece of human cognition

Fourteen articles conceptualized imagination as an important, higher order cognitive process or mental ability (Ayalew and Areaya, 2019; Chen and Yuan, 2021; Cheong et al., 2022; Frith et al., 2022; Gonzalez Garcia and Mukhopadhyay, 2019; Lin et al., 2014; Ness and Dysthe, 2020; Wu et al., 2017). Typically, imagination was viewed as an inherent cognitive process that varied through individual differences and an essential component leading to creative action or production (e.g., Dziedziewicz et al., 2013). This mental ability often relied on experiences (e.g., Chen and Yuan, 2021; Jankowska and Karwowski, 2015) and was frequently associated with making connections (e.g., Dereli, 2023; Horng et al., 2021; Ness and Dysthe, 2020), creating something new (e.g., Dziedziewicz et al., 2013; Ness and Dysthe, 2020; Richard et al., 2020), or helping the mind to conceive of something an individual has never experienced before (e.g., Kusmaryono and Maharani, 2021; Siswanto et al., 2020). According to von Stumm and Scott (2019), imagination “involves principal cognitive processes, such as perception and memory, that are often automated and only partly subject to effortful control” (p. 707) whereas Kaya and Acar (2019) implied that imagination was a “cognitive strategy” (p. 3). Seven of the studies carried out in Taiwan defined imagination as a cognitive or mental activity (e.g., Chen and Yuan, 2021; Horng et al., 2021) that served as the conductor and carried out the finer processes associated with imagination.

### 4.2 The role of imagination in the creative process

To explore the role of imagination in the creative process, we extracted the variables used to investigate the relationship as well as the findings from each article. For instance, in Ahmed's (2021) study, the extracted variables included an “imaginative approach,” which consisted of a visualization exercise and creative writing skills (p. 161). We also searched for any key passages in which authors addressed the relationship between imagination and creativity. This typically included passages from the discussion section in which authors elucidated on their findings. For example, Bauer and Gilpin (2023) reported that “the imagination–creativity link may depend on the level of self-regulation that a child has” (p. 7). See Appendix B for the data extraction form.

Intercoder agreement for variables used to explore the relationship between imagination and creativity was initially 97.6%, and remaining codes in question were discussed until reviewers reached consensus. All studies included in this review focused their exploration on creativity and imagination using a variety of variables. In this review, imagination was the most frequently used variable, occurring across all 42 studies, creativity occurred across 40 of the studies with the two exceptions examining creativity and imagination as a sort of blending of the two, or creative imagination (Frith et al., 2022) and imagination via initiation, fluency, flexibility, and originality (Lin and Tsau, 2013). Scholars often examined these constructs through tasks (*n* = 11), divergent thinking activities or assessments (*n* = 7), or creative thinking (*n* = 6) and typically used widely accepted indicators of creativity (i.e., originality [*n* = 12], fluency [*n* = 7], usefulness [*n* = 5], flexibility [*n* = 4]). Less common but still notable were variables related to creative potential (*n* = 3), academics (*n* = 3), creative visual imagination (*n* = 2), and semantic distance (*n* = 2). Scholars used these explorations to draw meaningful conclusions about the nature of imagination in the creative process.

Overall, imagination consistently predicted creativity (e.g., Chen and Yuan, 2021; Craft et al., 2013; Dereli, 2023; Grobelna, 2015; He et al., 2017; Horng et al., 2021; Joseph et al., 2023; Winarningsih et al., 2024) with three exceptions. Bauer and Gilpin (2023) found that imagination only predicted creativity when self-regulation was added to their model and both studies conducted by Fung and colleagues (Fung and Chung, 2022; Fung et al., 2021) noted their results as contrary to previous work in this area (i.e., imagination overexcitability was not related to creative potential). Although most supported the positive relationship between imagination and creativity, some authors pointed out some nuisances. For example, Zhang et al. (2024) found that imagination can enhance creative ideas but not the usefulness aspect. However, Craft et al. (2013) concluded that when used intentionally, imagination leads to original and valuable outcomes. Others identified imagination as a key mediator in the relationship with creativity (e.g., imagination as a mediator between personality and creativity, Hsu, 2019; between affective creativity and creative thinking, Wu et al., 2017). In another study, imagination was more strongly associated with creative ideation than creative ability (i.e., fluency, originality) and creative achievement (von Stumm and Scott, 2019). Kusmaryono and Maharani (2021) arrived at a similar conclusion: creativity was more closely associated with products whereas imagination was more closely associated with ideas and thinking. Although the relationship between these two constructs was consistently supported, imagination and creativity remained conceptually distinct.

#### 4.2.1 Related but distinct constructs

Many authors addressed imagination as a key component or an underlying basis of creativity, with 13 authors also clearly distinguishing between the two constructs. For instance, multiple authors explicitly characterized imagination and creativity as “highly related but distinct concepts” (Hsu, 2019, p. 310), “positively related [but] different cognitive processes” (Jankowska et al., 2019, p. 1112), and “two distinct psychological constructs” (Horng et al., 2021, p. 383). Across these studies, two dominant themes emerged: (a) imagination serves as the foundation of and often a precursor to creativity (*n* = 22), and (b) imagination is an internal process, whereas creativity is an external outcome (*n* = 13). See Table 5 for representative quotes for each of these themes.

**Table 5:** Related but distinct constructs.

Theme	Representative quote
Imagination serves as the foundation of and often a precursor to creativity.	“Imagination determines the scope of creative thinking and is the foundation of invention” (Chen and Yuan, 2021, p. 4).
	“Creativity is always said to involve imagination… Imagination is a necessary part of the human creative process (Williams and Walker, 2003). Based on these, it can be said that the wider the imagination of an individual, the more creative the individual can be (Gündoğan et al., 2013)” (Dereli, 2023, p. 159).
	“Although imagination and divergent thinking are key to a child's creativity, they engage partially different cognitive processes and refer to different material” (Dziedziewicz et al., 2013, p. 86).
	“Creativity springs from imagination. However, original ideas generated from imagination are only a precursor to creativity” (Horng et al., 2021, p. 376).
	“Imagination and creativity are two highly related but distinct concepts; they involve different operation processes and performance… imagination is the vehicle of active creativity; as a process, it creates ‘loops' out of the present, here-and-now of experiences and connects to ‘real' objects to conceive of and describe possible solutions” (Hsu, 2019, p. 310).
	“In general, a person's creativity is inspired by his or her imagination” (Hsu et al., 2014, p. 59).
	“The precondition of creativity is the autonomy of imagination (Kaplan, 1972)” (Liang and Lin, 2015, p. 271).
	“The imagination has been perceived as a central feature in human cognitive activity since the seventeenth century. Although its nature is unclear, it can be perceived as the basis for cultivating creative thinking, and thus, a driving force of innovation (Finke 1996)” (Lin et al., 2014, p. 73).
	“Thus, imagination serves as the basis of all creative activity, while all creative activity takes imagination to its full extent” (Lin and Tsau, 2013, p. 211).
	“Talking about imagination is very closely related to creative thinking activities (creativity), on the other hand talking about creative thinking (creativity) cannot be separated from imagination (Tsaniyah and Poedjiastoeti, 2017). Because creativity is the ability to produce something new and unique from the results of the thinking process (imagination) (Neto et al., 2019). Imagination is often said to be the basis of creative thinking activities” (Kusmaryono and Maharani, 2021, p. 290).
	“The creative process, in theory, begins with imagination, then moves on to improvisation, and finally, to a product with originality, something different and new” (Sungurtekin, 2021, p. 178).
	“Imagination is likely a precursor of creative ability, because original ideas may emerge as a result of recombining mental representations of ideas, concepts, and sensations” (von Stumm and Scott, 2019, p. 709).
	“Imagination was found to be a crucial element that a creator should both possess and develop… Creativity was generated by the imagination and direct experience via the other senses, including sight and touch (p. 599). Imagination is regarded as a prerequisite for musical creativity in a variety of different music activities (Hargreaves, 2012) and listening (Peterson, 2006)” (Zheng and Leung, 2021, p. 604).
Imagination is an internal process, whereas creativity is an external outcome.	“Researchers have observed that imagination refers to mental images of the creator, whereas creativity and creation are external displays of imagination” (Chen and Yuan, 2021, p. 4).
	“In this study, the distinction between imagination and creativity is based on whether students engage in deliberate action. In other words, imagination involves conceptualizing something that does not exist, whereas creativity involves creating something derived from an imagined concept” (Hsu et al., 2014, p. 59).
	“Creativity is the process by which a new, socially valuable product is produced. Whereas imagination refers to the mental process causing a new idea to emerge in the mind. While creativity produces something new to the material world; imagination produces something new in one's mental space” (Horng et al., 2021, p. 376).
	“The distinction between imagination and creativity in this study lies with whether the design student has an action. In other words, imagination is thinking of something that is not present, while creativity is doing something inventive with imagination” (Lin et al., 2014, p. 76).
	“Imagination denotes the ability to create new ideas, based on mental imagery such as true or fantastic elements, while creativity means the ability to interpret effectively and concretely some idea such as a song, a story, a product, which is like a critical thinking process” (Lin and Tsau, 2013, p. 218).
	“[Imagination] is also fundamentally different to creativity, which refers to the production of something that is novel and useful (Sternberg and Lubart, 1996), rather than to merely generating mental representations” (von Stumm and Scott, 2019, p. 707).
	“Imagination is the internal imagery of a creator whereas creativity and creations are the outward manifestation of imagination” (Zivkovic et al., 2015, p. 133).

Many authors agreed that imagination serves as a foundation of creativity, often assuming a precursory role to creativity (e.g., Chen and Yuan, 2021; Dereli, 2023; Eyada, 2022; Hsu et al., 2015; Kusmaryono and Maharani, 2021; Liang and Lin, 2015; Lin and Tsau, 2013; Liu et al., 2020; Ness and Dysthe, 2020; Puente-Díaz and Cavazos-Arroyo, 2017; Siswanto et al., 2020; Sungurtekin, 2021; von Stumm and Scott, 2019; Wu et al., 2017; Zheng and Leung, 2021; Zivkovic et al., 2015). One author described imagination as essential to the creative process, asserting that creativity always involves imagination (Dereli, 2023). Although not explicitly stated, Bačlija Sušić and Brebrić (2024) positioned imagination (alongside fluency) as a core feature of creativity in their study on creative musical expression. Moreover, imagination was often similarly described as a “catalyst for all creative actions” (Hsu et al., 2015, p. 588), the inspiration of one's creativity (Hsu et al., 2014, p. 59), and a source of “incubation for creativity” (Liang and Lin, 2015, p. 85), which underscored the vital role imagination plays as a conduit in the creative process.

Multiple authors elaborated on this distinction, viewing imagination as an internal process (e.g., mental imagery, idea generation) and creativity as an external outcome (e.g., action, performance, product). For instance, Chen and Yuan (2021) associated imagination with the “mental images of the creator” and creativity with the “external display of imagination” (p. 4). Similarly, Zivkovic et al. (2015) described imagination as “internal imagery” possessed by the creator whereas creativity is the “outward manifestation of imagination” (p. 133). Other scholars (e.g., Hsu, 2019; von Stumm and Scott, 2019) reinforced this distinction by conceptualizing imagination as a mental process and situating creativity within widely accepted product-oriented definitions that emphasize novelty and usefulness (Runco and Jaeger, 2012; Sternberg and Lubart, 1996). Horng et al. (2021) further clarified this distinction, explaining that:

Creativity is the process by which a new, socially valuable product is produced. Whereas imagination refers to the mental process causing a new idea to emerge in the mind. While creativity produces something new to the material world; imagination produces something new in one's mental space. (p. 376)

Lin and Tsau (2013) similarly positioned imagination in the ideation stage and creativity in the interpretative stage of the process (focusing on some type of creative product) whereas others simply pointed to action as the distinction between imagination and creativity (Hsu et al., 2014; Lin et al., 2014).

These quotes converged on the following point: imagination primarily operates as a process of ideation, and creativity involves the physical manifestation of those ideas. These patterns revealed authors' conceptualizations of imagination and creativity as closely related but functionally distinct constructs. This theme aligned with and expanded upon patterns that emerged in authors' definitions: imagination as an internal, generative process that typically involved mental imagery, and creativity as an external process in which ideas are transformed into observable action. Additionally, authors consistently placed imagination as the foundational precursor that drives creative action.

#### 4.2.2 Variables used to investigate the relationship between imagination and creativity

To further explore the role of imagination in the creative process, we examined the articles in our review to identify common variables examined alongside direct measures of imagination and creativity. Although most studies focused on creativity- and imagination-related variables, additional variables were also represented, with personality (*n* = 7) and psychosocial factors (*n* = 6) most used to explore the nature of the relationship between imagination and creativity.

##### 4.2.2.1 Personality traits

When studies included personality traits as main variables, studies reported significant relationships between personality traits and imagination (e.g., Hsu, 2019; Hsu et al., 2015; Liang and Lin, 2015; Liu et al., 2020; Smees et al., 2024). Some scholars adopted the Big Five model of personality (e.g., Hsu, 2019; Liang and Lin, 2015) with some limiting their investigation to a specific trait: openness (e.g., Grobelna, 2015; Taylor Bunce and Boerger, 2022; von Stumm and Scott, 2019). Others used creative personality (e.g., Horng et al., 2021; Hsu et al., 2015; Smees et al., 2024) of which a couple studies examined creative personality via affective creativity (e.g., Liu et al., 2020; Wu et al., 2017).

Of the Big Five personality traits, openness emerged as the most influential on imagination (e.g., Hsu, 2019; Liang and Lin, 2015; von Stumm and Scott, 2019). The remaining personality traits (extraversion, agreeableness, conscientiousness, emotional stability) had direct and indirect effects on imagination, especially when authors used the three-factor model of imagination (e.g., initiating, conceiving, transformation; Hsu, 2019). For example, Liang and Lin (2015) found that openness was linked to initiating imagination and flexibility. They also found that extraversion negatively predicted conceiving imagination whereas conscientiousness predicted conceiving imagination (Liang and Lin, 2015). In these studies, imagination was consistently linked to personality and creativity. The findings from these studies demonstrated three primary themes: (a) key personality traits, particularly openness, drive imagination and creativity (e.g., Liang and Lin, 2015; von Stumm and Scott, 2019); (b) imagination serves as a mediator between personality and creativity (e.g., Hsu, 2019; Hsu et al., 2015; Liang and Lin, 2015); and (c) when conceptualized as a personality trait, imagination predicts higher levels of creativity (e.g., Grobelna, 2015; Smees et al., 2024; Taylor Bunce and Boerger, 2022).

##### 4.2.2.2 Psychosocial variables

Multiple authors examined psychosocial variables in their investigations. Self-efficacy was explored as a potential predictor or mediator in five of the studies included in this review (e.g., Ahmed, 2021; Gonzalez Garcia and Mukhopadhyay, 2019). Findings indicated self-efficacy, including creative self-efficacy, as a positive predictor of both reproductive and creative imagination (e.g., Hsu et al., 2015; Puente-Díaz and Cavazos-Arroyo, 2017). Hsu et al. (2015) also suggested generative cognition and metacognition, described as inspiration through action, as significantly related to creative imagination, stating that this cognitive ability was likely “the most critical resource to connect human imagination and creativity” (p. 594). In another study, creativity was highest in children who had high levels of both imagination and self-regulation (Bauer and Gilpin, 2023). Additionally, Siswanto et al. (2020) found that affective creativity, defined as creative attitudes and emotional intelligence that facilitates creativity, moderated the relationship between imagination and creativity. In these instances, the relationship between imagination and creativity was evident, but the presence of these psychosocial variables seemed to heighten that relationship.

### 4.3 Toward a working definition of imagination

Collectively, these emergent themes and observed patterns of variable operationalization point to a cognitive ability that involves internal mental representations (imagery, visualizations), drawing on memories and experiences, and reconstructing or reshaping them to create something novel, future-oriented, or innovative. It is not just general thinking, but a mental process that bridges images and ideas, enables “what if” or possibility thinking, and allows individuals to move beyond their current reality to form new meanings, solutions, or creative outcomes. As a result, we propose the following definition:

Imagination is a higher-order cognitive ability that draws on prior experiences and uses mental imagery to activate, combine, and transform ideas, particularly in service of generating new possibilities, deepening one's understanding of potentialities, and facilitating the path to novel outcomes.

Importantly, this definition distinguishes imagination from creativity in that the latter typically carries with it an expectation of externalization and value. That is to say, ideas are not only imagined but expressed, evaluated, and situated within some context where their novelty is judged (along with meaningfulness and/or value; see Plucker et al., 2004). In that sense, imagination is best understood as a core precursor of creativity, but not all imaginative activity rises to the level of creativity until it is shaped, shared, and recognized in some consequential way. To further situate our definition, while imagination remains entirely internal—an unexpressed exploration of ideas—even creativity in mini-c form (see Kaufman and Beghetto, 2009) involves the construction of personally meaningful insights that are, in some form, externalized and articulated. In that sense, mini-c serves as a demarcation at which imaginative ability begins to take on defining features of creativity—even if only in the individual's own interpretive frame. Using idea generation—a feature that both constructs share—to frame or distinguish the constructs in this way highlights imagination as the starting point of idea generation whereas creativity represents what happens when those ideas are shaped, expressed, and eventually recognized or evaluated.

## 5 Discussion

Overall, the analysis of definitions and descriptions of imagination allowed us to formulate a more comprehensive view of imagination as a construct and its role in the creative process. In this review, emergent themes highlighted mental representations and imagery, transformation and transcendence of one's experiences, novel idea generation, and cognitive ability as key components of imagination. By defining imagination as a construct, the studies in this sample addressed what Shao et al. (2019) referred to as “the first order of business” when empirically investigating a new construct (p. 2).

In addition, authors who explicitly defined imagination and situated their definitions within pertinent contexts, such as domain and instrument, in turn responded to calls to move beyond implicit assumptions and conduct research that clearly situates creativity—and imagination, in terms of this review—within meaningful contexts (Forgeard and Kaufman, 2016; Plucker et al., 2004; Puryear and Lamb, 2025). More specifically, by connecting their operational definitions to the domain or instrument, researchers were able to identify important facets of imagination within a specific domain or uncover more complex relationships between imagination and creativity (e.g., reproductive imagination and film production, Hsu et al., 2015). This attention to context is critical for advancing empirical work in this area and avoiding pervasive debates that have long characterized creativity research (Hennessey and Amabile, 2010; Plucker et al., 2004; Puryear and Lamb, 2020; Runco and Jaeger, 2012; Simonton, 2016). Unlike scholarship in creativity, researchers made no argument for imagination as a domain specific construct; rather, situating imagination within a domain and relevant contextual factors provided a useful frame of reference for thematic patterns while also answering the question of imagination “for whom and in what context?” (Plucker et al., 2004, p. 92; Puryear and Lamb, 2020, p. 206; Puryear and Lamb, 2025).

Although authors' definitions provided some insight into the role of imagination in the creative process, a more granular view of this relationship emerged from the common variables used across studies in our review. Despite sharing some similarities with creativity, imagination was conceptualized as a distinct construct and identified as a significant predictor of creativity (e.g., Chen and Yuan, 2021; Dziedziewicz et al., 2013; Hsu et al., 2014; Kaya and Acar, 2019; Kusmaryono and Maharani, 2021; Lin and Tsau, 2013; Zivkovic et al., 2015). These findings empirically reinforce positions taken by other scholars who have attested to the important role imagination plays in creativity (Gotlieb et al., 2019; Stokes, 2014).

The identification of imagination as a precursor to creativity was also an important theme emerging from this review. Many scholars have devoted much of their time and effort to demystifying the creative process and improving our understanding of the conditions and contexts that support creativity (e.g., Amabile, 1983; Kaufman and Beghetto, 2009; Sawyer, 2021; Wallas, 1926). Identifying imagination, not just as a predictor of creativity, but as an essential precursor to creativity shows strong congruence with the Geneplore model (named by blending the terms generative and exploratory), particularly the generative phase. During this phase, mental representations, also called “preinventive structures,” can lead to creative discovery (Finke, 1996, p. 17). These preinventive structures operate as internal precursors to creative products that can be generated and modified through exploration. According to the Geneplore model, this process is not linear but instead functions as a cyclical process between both the generative and exploratory phases (Finke et al., 1996). Finke and colleagues' emphasis on mental representations as internal precursors to creativity also coincides with thematic patterns regarding conceptualizing imagination as an internal process and creativity as an external outcome. This also supports (Shao et al. 2019) assertion of creativity as a “consequence of human thought” (p. 4).

### 5.1 Limitations

This review was limited by the scope of the search and inclusion criteria. Restricting the search to peer-reviewed, English-language empirical articles published between 2012 and 2024 may have excluded relevant studies published in other languages or research disseminated in non-indexed outlets, such as conference proceedings or other reports not formally peer-reviewed. Additionally, although our aim was to capture imagination as it is conceptualized across contexts, the research-base remains somewhat underdeveloped with relatively few studies of imagination compared to creativity more broadly. As such, the patterns and themes identified here are subsequently shaped by what might be a limited or an uneven distribution of conceptions in research across disciplines, geographic-cultural regions, and methodological approaches.

In this review, a range of domains was represented (e.g., agriculture, art, engineering, math, music, science, teaching, writing), and many of the article authors provided essential information regarding imagination and related context. However, regardless of the specific domain utilized, imagination was routinely identified as a positive predictor of creativity. Importantly, many of these studies were correlational, and these results should be interpreted as representative of the studies included in this sample and not necessarily as an authoritative cause/effect relationship.

Approximately one quarter of the studies included in this review took place in Taiwan, which may represent a more Eastern or collectivist view of creativity and related constructs (Shao et al., 2019). However, thematic patterns were observed across studies from both Western and Eastern countries. For example, we noted that “imagination involves mental representations” appeared in about half of both Taiwan studies and studies as a whole. One theme that may be more culturally influenced is “imagination is an essential piece of human cognition” in which many supporting studies were conducted in eastern countries. Although themes were well-represented across cultures in this review overall, it is still important to consider that conceptions of creativity are culturally bound (Shao et al., 2019), and it is reasonable to consider imagination as similarly influenced by cultural context.

Regarding methodological approaches, 12% of the studies in this review used the ICS, which adopts a three-factor model of imagination (initiating, conceiving, transforming), to measure imagination. In these studies, authors offered a more nuanced account regarding how specific components of imagination related to creativity. For instance, initiating imagination predicted originality but not usefulness in creativity (Hsu, 2019). These studies also represented some of the mediator studies in our sample (e.g., Hsu, 2019; Lin et al., 2014). However, studies using the ICS were conducted in Taiwan, and while this did not affect the overall relationship between imagination and creativity that emerged in our findings, the concentration of this particular instrument within one geographical area may represent a confounding factor. This unevenness further suggests that our synthesis should be understood as reflecting the current, published research base rather than as a complete accounting of all possible perspectives on imagination and its role in the creative process.

### 5.2 Future directions and implications

We can apply many lessons from scholarly efforts to define creativity to empirically defining imagination. One such lesson includes investigating the ways culture may shape imagination as a construct (Shao et al., 2019). Moreover, because imagination emerged as foundational to creativity and as a precursor in the creative process, researchers must address this gap by considering imagination as an essential, vital component of the creative process. To be fair, creativity researchers have suggested imagination as a relatively important part of their personal conceptions of creativity they use in their work, though the degree of importance varies (Puryear and Lamb, 2025). Nonetheless, conducting more studies that examine the role of imagination in the creative process heeds the call placed by Forgeard and Kaufman (2016) and remains a fruitful area of exploration.

In this review, studies that used the ICS demonstrated strong validity and reliability; however, future research should conduct comparative analyses between the ICS and other instruments, such as the Imaginative Thinking Scale (ITS; Lin and Tsau, 2013). Also, since the ICS exhibited validity across domains and demographic characteristics, like age and gender, future studies should use other instruments, such as the TCIA (Jankowska et al., 2019), to measure imagination and examine whether other instruments are more appropriate for specific domains. Moreover, because studies using the ICS were limited to Taiwan, additional studies using the ICS should be carried out in other geographical locations, particularly with samples who may have a more Western view of creativity (Glǎveanu, 2018; Shao et al., 2019).

When investigating psychosocial factors, future research should more intentionally examine the role these factors play in shaping imaginative thinking and creativity, particularly how constructs such as self-efficacy and self-regulation can enhance or heighten imagination. Puente-Díaz and Cavazos-Arroyo (2017) similarly suggested future research that examines self-beliefs and their impact on imagery abilities rather than individuals perceived imaginative ability.

Other articles in this review point to a need for more research that explores additional relationships with imaginative capacities. For instance, researchers should consider investigating mediating and moderating effects on imagination (Liang et al., 2013), including the potential mediating role of cognition (Hsu et al., 2014). Expanding methodological approaches to include qualitative and mixed methods designs would also provide deeper insight into how imagination operates across contexts and populations (Dziedziewicz et al., 2014). As mentioned previously, both replication and exploratory studies should be conducted with more diverse populations to enhance the generalizability of findings in this area.

Additionally, other factors should be considered to improve our understanding of imagination as both a skill and a process. Personality traits, which have been shown to vary by discipline in both creativity and imagination research (Feist, 1998; Liang and Lin, 2015; Lin et al., 2014; Puryear et al., 2017), should be examined more closely in developmental studies within educational contexts. For instance, developing scientific imagination may vastly differ from the development of writers' or designers' imagination (Dereli, 2023; Gonzalez Garcia and Mukhopadhyay, 2019; Lin et al., 2014). Studying imagination through the lens of personality and discipline may better inform the design and implementation of talent development programs and discipline-specific learning experiences.

Environmental factors, such as social and school climate and teacher beliefs, also warrant further investigation in relation to the development of imagination in school-based settings. Research has supported the importance of these factors in supporting creativity in the classroom (Kettler et al., 2025). Increased attention in this area may help researchers to better understand how these factors may influence and support imaginative thinking (Chen and Yuan, 2021; Joseph et al., 2023) while also providing practitioners with evidence-based practices that support imagination, and thus, creativity. Investigating creative pedagogy and its effectiveness in developing and stimulating imaginative thinking could also be a promising area of research with important implications for educators. For instance, Chang et al. (2016) suggested research that investigates curricula that encourages students' entrepreneurial thinking and activity. Because teacher perceptions and beliefs play an important role in whether teachers desire creative characteristics and behaviors in the classroom (Kettler et al., 2018; Mullet et al., 2016), exploring teacher perceptions and beliefs about imagination and related constructs could also be beneficial (Sungurtekin, 2021).

From the sample in this review, additional recommendations for future research also included further elaborating imaginative capability in the creative process, with some scholars calling for studies that examine imagination in the creative genius (He et al., 2017). Except for Ness and Dysthe (2020), research on collective imaginative capability and its relationship to creativity was limited and remains an important area of further research. Additional suggestions included examining personality variables and their relationship to collective imagination (Liang and Lin, 2015). Although the relationships identified in this review enlightened and broadened our understanding of imagination and creativity, both independently and in concert, there remains substantial opportunity for continued exploration in this area.

## 6 Conclusion

Overall, emergent themes across studies provide a more coherent, empirically grounded understanding of imagination as a distinct yet integral component of the creative process. Imagination also emerges as an internal process that initiates and transforms ideas, enables possibility thinking, and facilitates creative action and outcomes. Based on our review, we propose a definition that situates imagination as a higher-order cognitive ability, emphasizes the key roles of mental imagery, prior experience, and the transformation of ideas, and preserves the distinctions between imagination and creativity. Moreover, positioning imagination as the starting point of idea generation offers a useful framework for understanding how creative outcomes are ultimately developed, expressed, and recognized. Moving forward, continued efforts to operationalize and investigate imagination within this framework will be essential for advancing theory, strengthening empirical work, and informing practices that intentionally cultivate imagination as a driver of creativity and innovation across contexts.

## Scope statement

Imagination is foundational in the creativity process; however, imagination receives little attention in creativity research. The purpose of this systematic review was to clarify imagination as a construct, examine its role in the creative process, and develop and propose an empirically grounded working definition of imagination. Findings revealed salient themes including imagination as an essential piece of human cognition, the driving force behind new ideas, a process involving mental representations, and the transformation of prior experiences to construct something new.

## Data Availability

The raw data supporting the conclusions of this article will be made available by the authors, without undue reservation.
