# Supplementary material for: Imagination and the creative process: a systematic review
Source: Front Psychol. 2026 Jul 3;17:1686856. doi: 10.3389/fpsyg.2026.1686856 (PMC13383033; doi:10.3389/fpsyg.2026.1686856)
Supplement: Supplementary file 1 [file Data_Sheet_1.pdf]

Appendix A

Screening Form with Eligibility Criteria

Eligibility Criteria	Yes/No
**Screening 1: Title/Abstract**	
**Abstract**: Does the abstract include both key terms (imaginat* AND creativ*).	
**Primary Variables:** Are imagination and creativity primary variables in the investigation?	
**Screening 2: Full-Text**	
**Study Type**: Is the study an empirical study with a QUAN, QUAL, or MM Method section?	
**Sample**: Is the sample described?	
**Study Purpose**: Do the authors address the relationship between imagination and creativity?	
**Decision: Include or exclude?**	
