# Supplementary material for: Imagination and the creative process: a systematic review
Source: Front Psychol. 2026 Jul 3;17:1686856. doi: 10.3389/fpsyg.2026.1686856 (PMC13383033; doi:10.3389/fpsyg.2026.1686856)
Supplement: Supplementary file 2 [file Data_Sheet_2.pdf]

Appendix B

Data Extraction Form

Article Descriptives

1. Database Name:
2. Journal Name:
3. Authors:
4. Article Title:
5. Year:

Study Descriptives

6. Method (Quan, Qual, MM):
7. Study Design:
8. Conceptual or Theoretical Framework (if provided):
9. Study Purpose:
10. Measure(s) Used:
11. Independent Variable(s):
12. Outcome Variable(s):
13. Domain (if applicable):
14. Location (Country):
15. Sample Characteristics:
16. Findings:

Research Question 1: Defining Imagination

17. Is imagination explicitly defined? (Yes/No). If yes, proceed to number 19.

18. If no, can a definition be inferred from the descriptions or characteristics provided (Yes/No). If yes, proceed to number 20.
19. Explicit definition offered (copy/paste definition and include page number):
20. Implicit definition provided, (copy/paste descriptions and include page number):
21. Copy/paste keywords/phrases used to explicitly or implicitly define imagination:

Research Question 2: The Role of Imagination in the Creative Process

22. Is the relationship between imagination and creativity addressed in the article? (Yes/No)
23. If yes, copy/paste excerpt(s) and include page number:
24. Does the author address imagination and creativity as distinct constructs? (Yes/No)
25. If yes, copy/paste excerpt(s) and include page number:
26. Reviewer Notes:

Note. This data extraction form was adapted to an Excel spreadsheet (e.g., each item number served as a column header. Reviewer 1 adapted the extraction form and pre-filled article descriptives only. Reviewers used the spreadsheet to independently extract and code each article.
